# Fundamental Limits to the Precision of Early Warning Systems for Epidemics of Infectious Diseases

**DOI:** 10.1371/journal.pmed.0020144

**Published:** 2005-05-31

**Authors:** John M Drake

**Affiliations:** University of California at Santa BarbaraSanta Barbara, CaliforniaUnited States of America

The development of early warning systems (EWSs) for epidemics of infectious diseases based on recurrent statistical patterns in other kinds of information, particularly data on climate, is an active area of research [1,2]. Judging from the estimated burden of diseases for which EWSs might be developed, such systems, if effective, would contribute greatly to human welfare and could potentially save many lives [2]. According to a recent report [2], EWSs have two principal aims: (i) to identify whether an epidemic will occur and (ii) to predict the number of cases that will result from it. For directly transmitted diseases, this second aim may be unattainable at the desired levels of precision, regardless of the quality of information.

As an example, in a recent report on the relationship between climate and outbreaks of meningococcal meningitis, the authors found that the timing of epidemics is highly predictable from information on the dynamics of a seasonal weather pattern, the Harmattan winds, but that the final epidemic size is not [1]. This finding is not surprising. The characteristics of disease outbreaks, particularly outbreaks of emerging diseases to which human populations are highly susceptible, prevent highly precise forecasts.

The reason that precise estimates of the final epidemic size cannot be obtained can be understood intuitively. Consider the following description of a typical outbreak. Characteristically, an outbreak begins with a small number of initially infectious individuals. Subsequent infectious contacts are mediated by a wide range of social interactions—contacts within and among households and communities—so that even individuals that are virtually identical can differ considerably in the number of secondary infections they cause. This is a micro-scale cause of variation compared with macro-scale, population-level sources of variation. The important implication for EWSs is that in such situations, especially where the basic reproductive ratio of infections (R0) is initially very high but is rapidly reduced (perhaps by public-health interventions), small deviations in the realized number of infectious contacts are amplified, resulting in relatively large variation in the final size of the outbreak. Because this variation reflects differences in individual behavior and not macroscopic characteristics of epidemic spread, it is unlikely that climate or other data contain any information about this source of variation (though such data do contain information about macroscopic variation).

A more formal explanation of this phenomenon can be formulated based on a simple model of an epidemic in which an EWS captures all macroscopic causes of variation in the final epidemic size but no microscopic causes. Obviously, an EWS cannot realistically be expected to capture even all the macroscopic information. Thus, this limit to precision is a fundamental limit and should be interpreted as a theoretical upper bound on forecast precision. The simplest case considers a disease with only two macroscopic epidemiological characteristics, an infection rate and a removal rate, which may change over time as in the case of meningococcal meningitis. In particular, we assume that there is no immunity in the population and that infection and removal are independent in time. This model of disease dynamics belongs to a class of stochastic processes known as nonhomogenous birth–death processes, which, conveniently, turn out to be reasonably tractable. More than 50 years ago, Kendall [3] showed how models for the mean and the variance in the final epidemic size are affected by these parameters. The variance can be interpreted as a measure of the precision with which the final epidemic size can be predicted. Kendall's results can be broken down to show that this quantity is equal to the sum of the average final epidemic size and another quantity (x) minus one. For most realistic epidemiological parameters, this other quantity, which is related to the covariance between final epidemic size and the size of the infected population, will be much greater than one. In these cases, the variance in the final epidemic size will be much greater than the average final epidemic size itself.

This fundamental limit to the precision of forecasts does not imply that EWSs cannot be used effectively to plan a response to outbreaks. Rather, it suggests what expectations of EWSs are reasonable. Further, since the precision with which forecasts of the final epidemic size can be obtained will depend on many disease-specific properties and maybe other factors, too, case studies of the potential effectiveness of EWSs for different diseases are needed. These studies should exploit recent advances in modeling birth–death processes [4] to gain further understanding of the differences among diseases and of the causes of geographic variation in the intensity of epidemics. Finally, notwithstanding limits to precision, the benefits to be obtained from estimates of the average final epidemic size and the timing of epidemics alone may warrant considerable investment in EWSs.
